# Socially vs. Privately Optimal Control of Livestock Diseases: A Case for Integration of Epidemiology and Economics

**DOI:** 10.3389/fvets.2020.558409

**Published:** 2020-11-25

**Authors:** Ângelo J. Mendes, Daniel T. Haydon, Emma McIntosh, Nick Hanley, Jo E. B. Halliday

**Affiliations:** ^1^College of Medical, Veterinary and Life Sciences, Institute of Biodiversity, Animal Health and Comparative Medicine, University of Glasgow, Glasgow, United Kingdom; ^2^College of Medical, Veterinary and Life Sciences, Institute of Health and Wellbeing, University of Glasgow, Glasgow, United Kingdom

**Keywords:** livestock, infection, disease control, model, epidemiology, economics

## Abstract

This paper aims to illustrate the interdependencies between key epidemiological and economic factors that influence the control of many livestock infectious diseases. The factors considered here are (i) farmer heterogeneity (i.e., differences in how farmers respond to a perceived disease risk), (ii) off-farm effects of farmers' actions to control a disease (i.e., costs and benefits borne by agents that are external to the farm), and (iii) misalignment between privately and socially optimal control efforts (i.e., privately optimal behavior not conducive to a socially optimal outcome). Endemic chronic diseases cause a wide range of adverse social and economic impacts, particularly in low-income countries. The actions taken by farmers to control livestock diseases minimize some of these impacts, and heterogeneity in those actions leads to variation in prevalence at the farm level. While some farmers respond to perceived disease risks, others free-ride on the actions of these individuals, thereby compromising the potential benefits of collective, coordinated behavior. When evaluating a plausible range of disease cost to price of control ratios and assuming that farmers choose their privately optimal control effort, we demonstrate that achievement of a socially optimal disease control target is unlikely, occurring in <25% of all price-cost combinations. To achieve a socially optimal disease control outcome (reliant on farmers' voluntary actions), control policies must consider farmer heterogeneity, off-farm effects, and the predicted uptake of control measures under the assumption of optimized behavior.

## Introduction

Livestock production is vital to economic development in many low-income countries, contributing to poverty alleviation, investment in children's education, and food security (1, 2). Infectious diseases jeopardize these societal functions, yet their control has become increasingly reliant upon privately funded actions (3–8). This “voluntary approach” to disease control distributes costs and responsibility between private agents (farmers that invest in control measures) and the government (animal health authorities that promote and often regulate farmers' actions) (9). The problem of the voluntary approach is that self-interested behavior can fail to produce a socially optimal level of disease control (i.e., the level of disease control that maximizes the sum of all agents' payoffs in society, the social optimum) (8, 10). The market thus “fails” to achieve the social optimum.

One reason for such “market failure” in disease control is the presence of externalities: costs and benefits borne by agents who are not parties to a market transaction (8). Externalities can be positive (e.g., a farmer enjoys a lower risk of disease incursion into his herd when neighboring farmers vaccinate their animals) or negative (e.g., a farmer has a higher risk of disease incursion into his herd when other farmers sell sick animals into a livestock market). One farmer's investment in animal health can thus influence other farmers' levels of disease risk. The way individual farmers perceive and act upon their level of risk may fail to produce a social optimum: some farmers free-ride on the control efforts of others, and the potential benefits of one farmer's actions can be limited by others' inaction (9, 11). Achieving the socially optimal level of disease control often requires governments to intervene (e.g., through financial incentives and regulation) in a way that accounts for the nature of farmers' decision-making processes and the interdependencies between farmers' choices and disease dynamics (3, 8, 9, 12–19).

The way farmers perceive and respond to disease risks is not only affected by disease spread but also impacts upon it. There are feedback processes between how a disease spreads and the responses of farmers to changing risks (20–22). When the inter-dependent dynamics of economic, behavioral, and epidemiological factors are not understood or well-represented in models of disease transmission, then policies aimed at promoting farmers' action may have perverse effects (23–26).

Our objective in this paper is to illustrate interdependencies between key epidemiological and economic factors that are not consistently taken into account in models of livestock diseases: (i) farmer heterogeneity (i.e., differences between farmers in how they respond to perceived risks); (ii) associated externalities; and (iii) misalignment between privately and socially optimal control efforts. We demonstrate the impact of farmer heterogeneity on disease prevalence over time. We quantify the externalities associated with farmer heterogeneity and show how the outcome of any individual farmer's control effort depends on the behavior of others. Finally, we examine a farmer's decision in trading-off *ex ante* disease prevention expenditure and *ex post* treatment expenditure and output loss, to estimate the probability that adoption of privately optimal behavior will achieve a socially optimal disease control target. The consequences of a socially sub-optimal control level depend on, among other factors, the population at risk. Here, for simplicity and to illustrate the general case, groups of farmers represent society.

## Methods

We explore the relationships between epidemiological and economic factors using a deterministic compartmental model (27). The model assumes a chronic infectious disease of livestock with a low basic reproduction number (R_0_ = 1.11), a livestock species with an average lifespan of five time units, and a constant herd size over time (250 animals on farm). We apply the model in three scenarios that explore farmer heterogeneity, quantify externalities, and examine differences between privately and socially optimal control efforts. In all scenarios, the animal-level prevalence in each farm starts at 10% (the endemic equilibrium, i.e., when prevalence remains steady and the effective reproductive number, R_e_, is 1.00). We simulate the time required to achieve a prevalence of <2% following a hypothetical awareness-raising event (e.g., information provision at time zero) and a time horizon of 25 time units. This target prevalence of <2% represents an economic optimum from society's perspective (i.e., the socially optimal level of disease control). A 2% threshold can also be considered a prevalence level below which costly government-led strategies aimed at eliminating the disease (e.g., test-and-slaughter) are required (28). In our simulations, for simplicity and without loss of generality, we assume infected animals show chronic symptoms and lose productivity, but there is no disease-associated mortality or infertility. Individuals can be either susceptible or infected; once infected, they remain infected and infectious for the remainder of their lives. Complete recovery from the disease is not possible.

The model set-up is as simple as possible for demonstration purposes. The basic features are (i) two states (susceptible—*S*; and infected—*I*), (ii) continuous time steps, (iii) equal birth and death rates (α = μ= 0.20), and (iv) an initial transmission rate (β) of approximately 8.89 × 10^−4^, reflecting the endemic equilibrium. The model can be run with one or more farms, and disease transmission can occur within farm only or both within and between farms. The proportion of the initial overall β attributable to between-farm transmission (q) is 0.0 when there is no transmission between farms, and 0.1 when between-farm transmission is included. The amount of control effort applied on farm is represented by a proportionate reducer on β ($r_{i}^{\beta }$). The model's differential equations are as follows:

(1) $$\begin{array}{l} \frac{\text{d}S_{i}}{\text{dt}}=\alpha \left(S_{i}+I_{i}\right)-\left(1-\text{q}\right)\left(1-r_{i}^{\beta }\right)\beta S_{i}I_{i}\\ \;-\text{q}\beta S_{i}\overset{n}{\underset{\begin{array}{c} j=1\\ j\neq i \end{array}}{\sum }}I_{j}-\;\mu S_{i} \end{array}$$

(2) $$\begin{array}{r} \frac{\text{d}I_{i}}{\text{dt}}=\left(1-\text{q}\right)\left(1-r_{i}^{\beta }\right)\beta S_{i}I_{i}+\text{q}\beta S_{i}\overset{n}{\underset{\begin{array}{c} j=1\\ j\neq i \end{array}}{\sum }}I_{j}-\;\mu I_{i} \end{array}$$

The differential equations were solved in *R* (29) (version 3.6.2) with the package *deSolve* (30) (version 1.27.1). All plots were generated using the *ggplot2* package (version 3.3.0) (31).

We demonstrate the effect of farmer heterogeneity on the predicted prevalence of disease over time by simulating transmission-reducing measures in three farms following a hypothetical awareness-raising event. As farmers continually assess the benefits and costs of their actions over time, the amount of control effort in each farm is set to be a function of disease prevalence (*p*_*i*_). We assume that (i) disease transmission occurs within each farm only, and (ii) as the prevalence (and disease impact) at the farm declines, the control effort applied ($r_{i}^{\beta }$) also falls (32), as follows:

(3) $$\begin{array}{r} r_{i}^{\beta }\;=\;\frac{1}{1\;+{\text{e}}^{-100\left(p_{i}-\gamma_{i}\right)}} \end{array}$$

where γ_*i*_ represents the farmer responsiveness. γ was set at 0.025, 0.100, and 0.175 for the farmer that is “highly responsive,” “moderately responsive,” and “slightly responsive,” respectively. Note that, as the prevalence decreases in any farm due to the control effort applied, the farmer responsiveness does not change—it is a characteristic of each individual farmer.

We then estimate the off-farm effects (externalities) of livestock disease control in two of the above farms: one in which the farmer is highly responsive and the other in which the farmer is slightly responsive. Firstly, we quantify the *ex post* cost of disease over time at the farm level by assigning a monetary value (δ) to the cost of each infected animal per time step. We assume that the *ex post* cost of the disease varies linearly with prevalence (i.e., the cost per time step is the product of the number of infected animals and the cost per infected animal). We then compare the *ex post* cost of disease over time in each farm when the transmission rate between farms (bβ) is 0% (q is 0.0) and 10% (q is 0.1) of the initial within-farm transmission rate (wβ). Actions taken by each farmer to reduce transmission in their farms only reduce the wβ—and do not influence bβ, which is kept constant over time. By holding the bβ constant over time and at a common value for the two farms, we demonstrate the off-farm effects of on-farm actions only—this assumes that (i) the contact rate between farms is kept constant, and (ii) the force of infection is proportional to the number of infected animals on each farm. Variation in farm number and density is unlikely to affect the qualitative inferences from this simulation.

We define “cost of disease” as the sum of disease-related output loss (production potential that is not realized over time) and expenditure on treatment of infected animals, plus the cost of control. Treating infected animals does not affect their infectiousness. Cost of control refers to money spent on reducing the wβ to prevent new cases, for instance, through improvements in hygiene and biosecurity. The cost of disease can thus be split into *ex ante* cost (cost of control) and *ex post* cost (disease-related output loss and treatment-related expenditure).

We demonstrate the market failure in livestock disease control by comparing the private net profit of farmer *i* (*Π*_*i*_) with the social net profit (*Π*_*s*_), the sum of the private net profits of *n* farmers ($\Pi_{s}=\sum_{i=1}^{n}\Pi_{i}$). *Π*_*i*_ denotes the private net profit of farmer *i* for the whole simulation period of 25 time units ($\Pi_{i}=\overset{25}{\underset{t=1}{\sum }}\Pi_{it}$). The example has only two farmers for simplicity, so *Π*_*s*_ = *Π*_*R*_ + *Π*_*r*_; the social net profit is the net profit of the highly responsive farmer (*R*) plus the net profit of the slightly responsive farmer (*r*). Considering that the benefit (*b*_*i*_, the number of animal infections averted) enjoyed by the highly responsive farmer as a result of disease control effort is a function of control effort applied in their farm ($r_{R}^{\beta }$) as well as in the other farm ($r_{r}^{\beta }$) (i.e., $b_{R}=f\left(r_{R}^{\beta },r_{r}^{\beta }\right)$), the net profit *Π*_*Rt*_ (in time step *t*) is given by $\delta b_{R}-\theta r_{R}^{\beta }$, where δ is the *ex post* cost per infected animal (treatment expenditure and output loss), and θ is the unit price of control action (the price of reducing the transmission rate by 1%). For this simulation, we assume that δ and θ are fixed at 50 and 0.10 monetary units, respectively.

We examine the effect of optimizing behavior on disease prevalence by assuming that the farmer reduces the wβ so that the total cost of the disease (the sum of *ex ante* and *ex post* costs) at each time step is minimized (32). This process is repeated iteratively for each level of the unit price of control (θ, ranging from 0.00 to 1.25 monetary units per time step) and *ex post* cost per infected animal (δ, ranging from 10 to 85 monetary units per time step). We assume (conservatively) that (i) the farmer has perfect information of both the *ex ante* and *ex post* costs of disease, and (ii) their behavior is no longer determined by a pre-specified logistic function. Instead, at each time step, the farmer reviews and optimizes their control effort given the prevalence. The short planning horizon (one time step, equivalent to one-fifth of the animals' average lifespan) reflects the generally high time-preference rates in low-income countries (33).

The qualitative inferences drawn from the model outputs are observed under a range of alternative scenarios and assumed parameter values. Readers can explore different scenarios and assumptions in the interactive web application, which is available here: http://boydorr.gla.ac.uk/eemodel/epiecon. This application was developed with *shiny* (34) (version 1.4.0) and *shinydashboard* (35) (version 0.7.1) packages in R (29). The R script for all the simulations is available at: https://doi.org/10.5281/zenodo.4108335.

## Results

### The Impact of Farmer Heterogeneity

**Figure 1:**
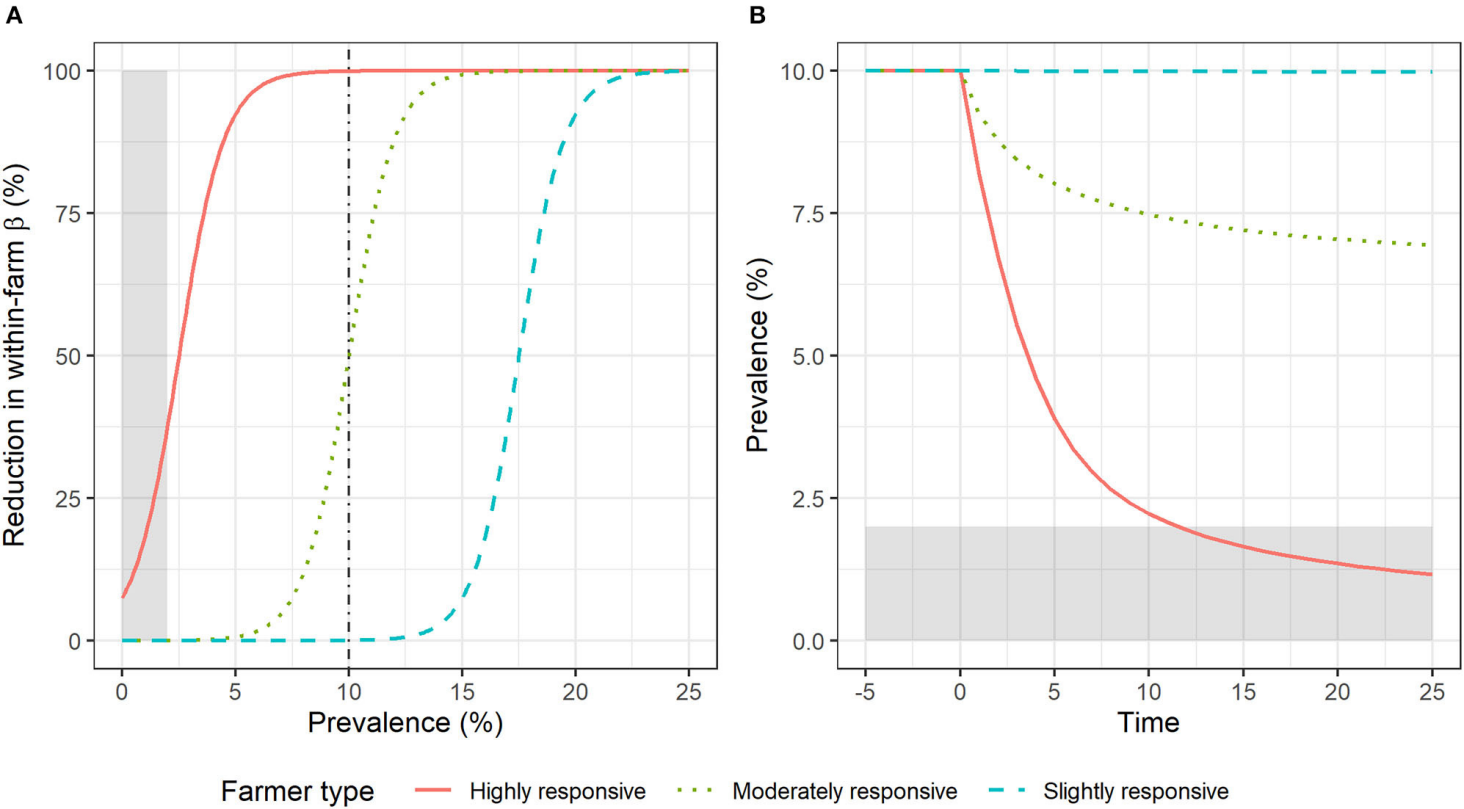
Effect of farmer type on disease prevalence following a common starting prevalence and a hypothetical awareness-raising event (at time zero). **(A)** Control effort applied (percentage reduction in transmission rate—β—within the farm in relation to the initial β in endemic equilibrium) as a function of disease prevalence for three different farmer types. The vertical dash-dotted line indicates initial prevalence. **(B)** Disease prevalence over time within the three farms. In both panels, the gray shaded area indicates the target prevalence level from society's perspective (<2%).

The prevalence of the disease is estimated over time within three model farms managed by three different “types” of farmers who vary in their responsiveness to an awareness-raising event. The responsiveness level of each farmer type is given by a behavioral parameter (γ_*i*_). The model uses this parameter in a logistic function that determines the reduction in transmission rate caused by each farmer's different actions at any given level of prevalence (Figure 1A). For instance, at the initial prevalence of 10%, (i) the highly responsive farmer reduces transmission by 100% (red solid line), (ii) the moderately responsive farmer reduces transmission by 50% (green dotted line), and (iii) the slightly responsive farmer does not reduce transmission at all (blue dashed line). The impacts of these three levels of responsiveness on disease prevalence over time are shown in Figure 1B. The heterogeneity in farmer responsiveness has an impact on the level of control effort applied and thus on the reduction in prevalence achieved. Among the three farmer types, only the highly responsive type achieves the target prevalence (i.e., <2%) within 25 time units.

### Externalities: The Off-Farm Effects of Farmers' Control Actions

Now we model disease prevalence in two neighboring farms (managed by highly and slightly responsive farmers, respectively), allowing for between-farm transmission, to explore the off-farm effects of farmers' control actions upon within-farm and societal costs of disease, represented by the sum of costs over the two neighboring farms.

**Figure 2:**
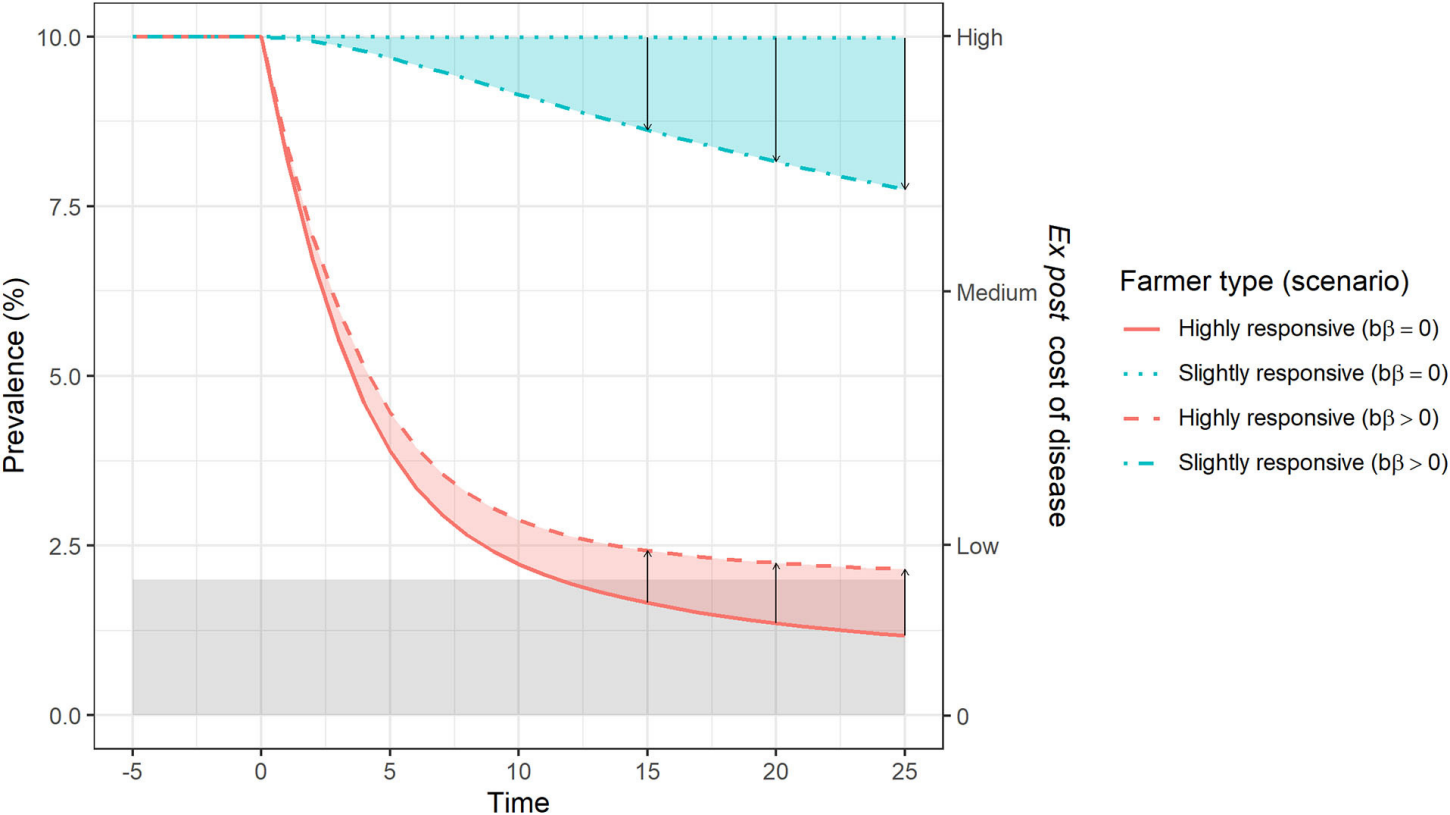
Externalities occurring as a result of different effort to achieve disease control in two neighboring farms. Arrows indicate a simulation scenario shift, from absence of transmission between farms (bβ = 0) to a situation in which between-farm transmission is possible (bβ > 0). Assuming an *ex post* cost per infected animal per time step (δ) of 50 monetary units, the labels “Low,” “Medium,” and “High” on the right vertical axis correspond to 313, 781, and 1,250 monetary units. Blue and red shaded areas correspond to the positive and negative externalities, respectively. The gray shaded area indicates the target prevalence level from society's perspective (<2%).

For each farm, we estimate the *ex post* cost of disease over time following an awareness-raising event. Figure 2 shows the estimated *ex post* cost in two farms with and without between-farm transmission. With no off-farm effects (i.e., bβ = 0, and thus no effects of neighbor's actions on costs), a highly responsive farmer (solid red line) reduces prevalence and *ex post* cost. In contrast, for a slightly responsive farmer (dotted blue line), the prevalence and the *ex post* cost are unchanged over time. When the off-farm effects or externalities are included (i.e., bβ > 0, transmission between farms is possible), the highly responsive farmer sees a reduced benefit of their actions, and the slightly responsive farmer benefits from their neighbor's actions (compared to the equivalent costs when bβ = 0). The extent by which the first farmer (highly responsive) reduces the prevalence of disease (and its associated cost) is limited by the force of infection originating from the second farm, where the farmer is only slightly responsive. Also, despite the lack of control action, the prevalence and *ex post* cost of disease at the second farm drops over time as a result of a decreasing force of infection originating from the first farm, where the prevalence is decreasing due to the control measures applied. Using a discount rate of 5%^1^, the extent of market failure^2^ is the difference between the $\Pi_{\;}^{s}$ (849 monetary units) and $\Pi_{\;}^{R}$ (695 monetary units). This difference is due to the benefits enjoyed by the slightly responsive farmer, who does not incur any costs of control.

### Privately Optimal Behavior and Reductions in Prevalence

**Figure 3:**
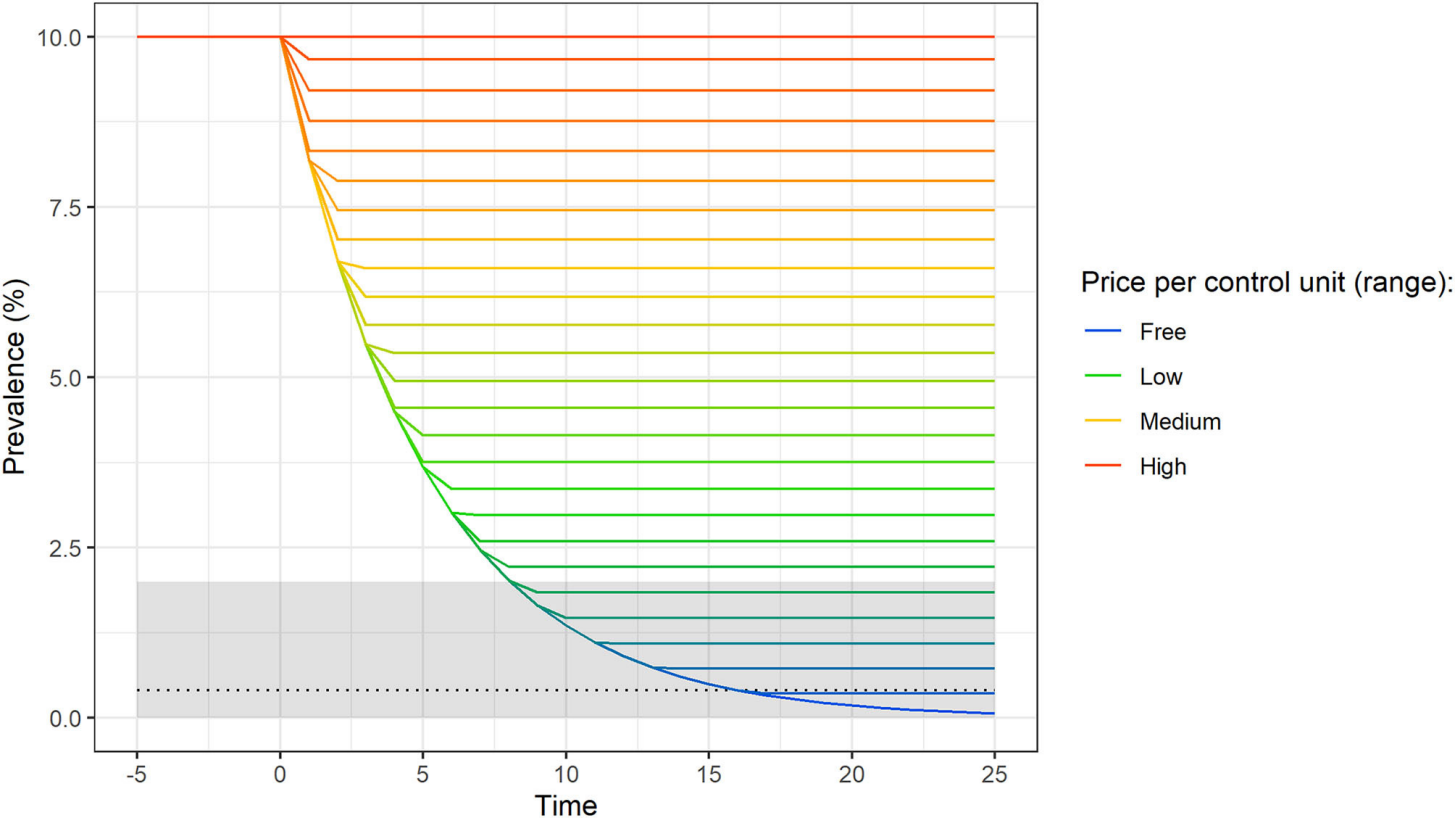
Disease prevalence over time, assuming privately optimal behavior in a single farm for a range of prices per control unit. This simulation assumes a cost of one infected animal per time step (δ) of 50 monetary units. The price of reducing the transmission rate by 1% (θ) ranges from “Free” (0.00 monetary units) to “High” (1.25 monetary units). “Low” and “Medium” correspond to 0.40 and 0.85 monetary units, respectively. Colored lines represent increases by 0.05 monetary units. The gray shaded area indicates the target prevalence level from society's perspective (<2%). The black dotted horizontal line indicates a prevalence consistent with disease elimination (0.4%; <1 infected animal in a herd of 250 animals).

Here we model disease prevalence (with a single farm and bβ = 0) to establish if privately optimal actions of a farmer will achieve the socially optimal target prevalence (i.e., <2%). The prevalence over time assuming privately optimal behavior for different unit prices of control (from “free” to “high”) is shown in Figure 3. Following an awareness-raising event (at time zero) and assuming a fixed *ex post* cost per infected animal (δ= 50 monetary units), the extent by which the within-farm prevalence is reduced varies inversely with the unit price of control (θ). Achieving the socially optimal target prevalence is possible if the unit price of control is very low. Disease elimination (prevalence below 0.4% or <1 infected animal in a herd of 250; note that the predicted prevalence is strictly continuous) only occurs when the unit price of control is equal or <0.05 monetary units (first line above “free” in Figure 3). When the unit price of control is above 0.05 monetary units, the farmer's optimal control effort either reduces the prevalence until it reaches a new endemic equilibrium or does not reduce it at all (shown as the uppermost flat line in Figure 3; the prevalence remains at the initial endemic equilibrium level). This model of optimizing behavior shows that self-interested farmers, who optimize their private investment in disease control, as assumed here, may not achieve the target prevalence from society's perspective in the absence of an intervention by, for example, the government.

In our simulations, under the assumption of optimizing farmer behavior, as prevalence drops, the marginal cost of control actions (i.e., the cost of reducing the prevalence by an additional unit, 1%), increases sharply. It is more expensive to reduce the prevalence from 3 to 2% than from 10 to 9% because the number of cases prevented per time step by reducing the transmission rate depends on the number of currently infected individuals. If the number of currently infected individuals is high (e.g., 10% prevalence), the prevention of new cases yields a quick reduction in prevalence. If the number of currently infected individuals is low (e.g., 3% prevalence), further reductions of the prevalence rely to a greater extent on the elimination of currently infected individuals. Under the assumption of optimizing behavior, as the marginal costs of control increase, the farmer's investment in control therefore gradually declines until an equilibrium is reached in both epidemiological and economic terms (shown as the plateau for each line in Figure 3). This equilibrium prevalence level is reached when the *ex ante* expenditure in one time step equals the averted *ex post* cost of disease.

**Figure 4:**
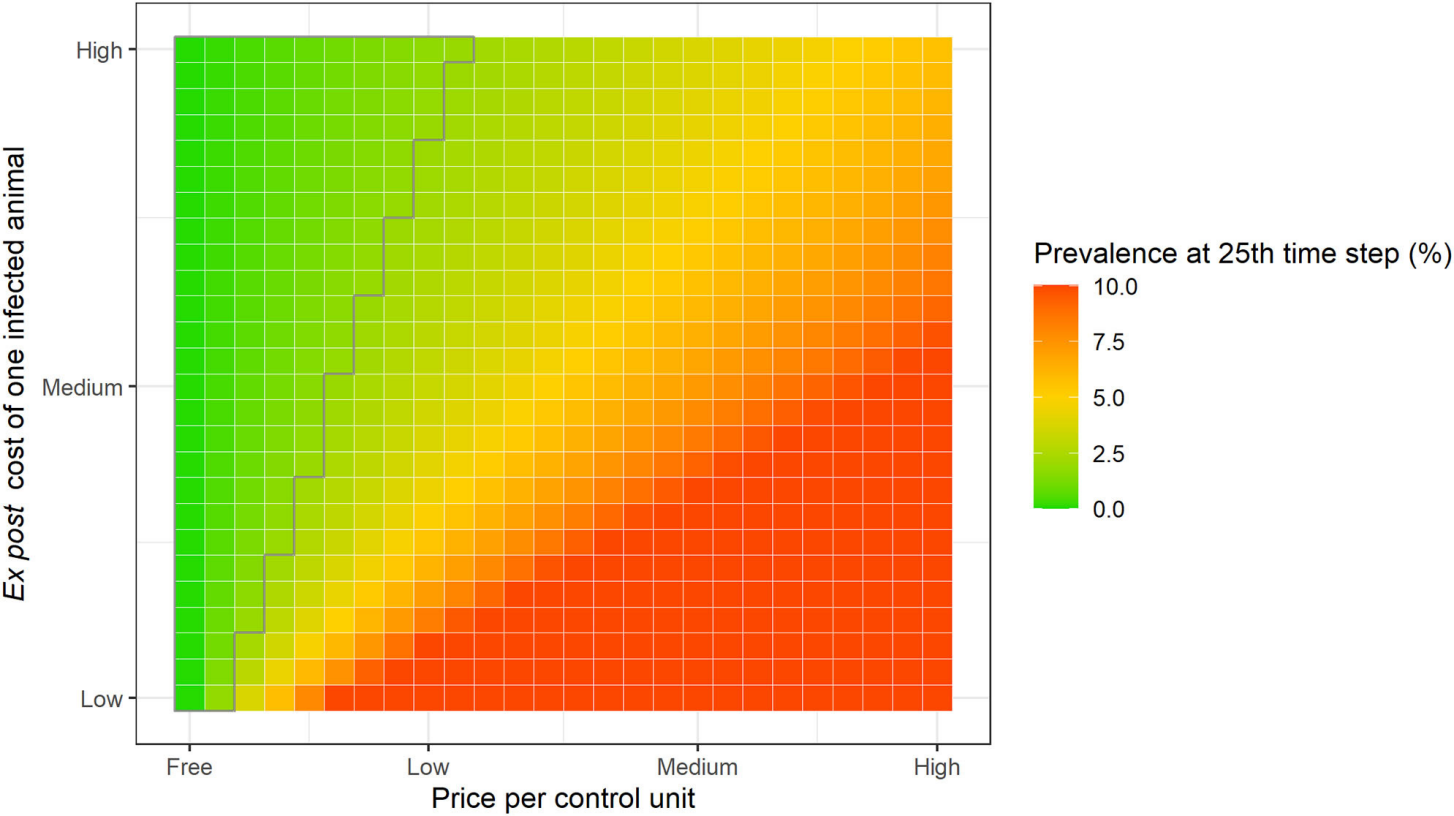
Predicted prevalence at 25th time step for different values of price per control unit (“Free” = 0.00; “Low” = 0.40; “Medium” = 0.85; and, “High” = 1.25 monetary units) and *ex post* cost per infected animal (“Low” = 10; “Medium” = 46; and, “High” = 85 monetary units), assuming privately optimal behavior in a single farm. The polygon with gray border indicates the target prevalence level from society's perspective (<2%).

The achievement of a socially optimal outcome prevalence depends on the specific values of the unit price of control and cost per infected animal selected. The results shown in Figure 3 considered a fixed *ex post* cost of one infected animal (δ= 50 monetary units). However, for the same disease and animal species, this value could in practice vary with the price of livestock, cost of production inputs, type of production system, etc. Figure 4 shows the predicted prevalence after 25 time units for a range of values of price per control unit and a range of values of *ex post* cost per infected animal. When we allow the cost per infected animal to vary in plausible ranges in relation to the price of control unit (e.g., eliminating disease transmission by 100% would cost a maximum of 125 monetary units per time step, which is <1.5 times the highest *ex post* cost of one infected animal, i.e., 85 monetary units), the previously observed pattern holds: farmers who optimize their control effort may not achieve the target prevalence from society's perspective.

## Discussion

Analytical frameworks introducing human behavioral dynamics in the field of livestock infectious disease modeling have emerged in the last two decades. From the pioneering works of Bicknell et al. (24) and McInerney et al. (32) to the most recent individual-based, network-based, and game-theoretic models (9, 36–38), many significant contributions have improved our understanding of the interplay between human behavior and livestock disease spread (and the likelihood of disease control). However, this study is unique and contributes to that body of knowledge for two reasons. Firstly, it provides a simple but robust framework for exploring key factors in the control livestock diseases: farmer heterogeneity, externalities, and private-social misalignment of control optima. Secondly, we apply this framework to the control of endemic chronic diseases through privately funded actions in a low-resource setting.

Despite the growing literature on livestock infectious disease modeling, very little attention has been given to the assumption of homogeneous human behavior, the validity of which we evaluate here. Models of livestock disease typically assume that the farmers' perception of risk and the way they respond to that level of perceived risk remain unchanged over time (22). In addition, many epidemiological models entirely ignore the effects of changing prevalence on privately optimal spending on disease control (22, 23). However, several empirical studies have shown that a farmer's likelihood to take control actions is the result of a dynamic and complex interplay between epidemiological, economic, environmental, cultural, and social factors (39–43). Models of disease transmission that ignore such heterogeneity are likely to be misleading, resulting in reduced value for informing control efforts. Like many decision-makers, farmers make decisions based on conscious and unconscious, cognitive and affective shortcuts or rules of thumb (42, 44, 45). We model the implications of this type of individual decision-making process by simulating the effects on prevalence and costs of different levels of farmer responsiveness. These simulations quantify the consequences of heterogeneous behavior, the occurrence of externalities, and the consequent disparities between privately and socially optimal control efforts.

The simulations of our theoretical scenario of optimizing behavior assumed perfect information and rational behavior by the individual farmer. In this scenario, the farmer was able to accurately choose the optimal control effort at any point in time by trading-off the marginal benefits of reducing the prevalence against the marginal costs of their actions. These models assume a conservative range of values for the unit price of control action (from 0.00 to 1.25 monetary units to reduce the transmission rate by 1% per time step) and cost of one infected animal (from 10 to 85 monetary units per time step). For example, without administration and overhead charges, the annual cost of vaccinating a herd of 250 cattle for brucellosis in India (167.50 USD; 0.67 USD times 250) would be over twice as much the average loss caused by brucellosis per infected animal (73.44 USD) (46). The values that we used are considered appropriate and conservative for this evaluation because the highest possible cost of eliminating disease transmission completely would be equivalent to <1.5 times the highest *ex post* cost of one infected animal per time step. Despite these conservative assumptions, the societal disease control target was only achieved for high disease cost to price of control ratios (<25% of all price-cost combinations; Figure 4). Given these findings, we can infer that effective disease control through voluntary actions is unlikely to be privately optimal in many real-world cases. Additionally, as prevalence decreases, there is a sharp increase in the marginal costs of control action, which leads to a fall in optimal control effort. This result is consistent with the economic principle of diminishing returns and has also been reported by other theoretical (32) and empirical studies, namely those that analyzed control actions against tuberculosis (24, 47) and brucellosis (48, 49) in cattle.

Our model illustrates the misalignment of private and social optima for livestock disease control through a basic trade-off between the benefits of reductions in herd prevalence and the costs of transmission-reducing actions. Essentially, farmers “buy reductions in disease transmission,” which is a simple and logical representation of many disease control actions. However, this approach is likely to underestimate the gap between private and social optima in many cases: the model does not capture many of the benefits of farmers' actions such as (i) the control of multiple pathogens through improved biosecurity, and (ii) the human health benefits in the cases of zoonoses. For instance, brucellosis is a bacterial zoonosis that causes abortion in livestock and debilitating symptoms (e.g., fever, joint pain, myalgia) in humans (50, 51). Given this burden on human health, in addition to animal health, voluntary actions taken by farmers to control the disease in their livestock (e.g., through vaccination) generate positive externalities, as society benefits from farmers' actions whilst not bearing the costs of these actions. This is a market failure that requires coordinated action to achieve socially optimal outcomes in terms of both human and animal health and productivity (48). Hence, the benefits enjoyed and the costs born by both public and private sectors must be considered throughout the planning phase of control interventions, which can include, among others, the provision of subsidized goods and services (e.g., vaccination), creating and enforcing regulations (e.g., movement restrictions) and setting compensation schemes (e.g., for culled seropositive animals) (8, 48).

To enable private farmers' actions to achieve a socially optimal disease control target, policy design and development must consider the heterogeneity of farmer behavior, the associated off-farm effects or externalities, and the predicted uptake of control measures under optimized farmer behavior. Failure to acknowledge these factors may result in potentially misleading predictions about disease transmission and the associated economic costs. This, in turn, perpetuates conditions in which private investments by farmers are inadequate to achieve disease control from society's perspective. For multi-host diseases that can impact multiple sectors (e.g., multi-host livestock diseases and zoonoses), inadequate disease control can have profound repercussions on international trade and, indeed, threaten human health.

## Data Availability Statement

The original contributions presented in the study are included in the article/supplementary materials, further inquiries can be directed to the corresponding author/s.

## Author Contributions

ÂM, DH, EM, NH, and JH contributed to conception and design of the study and writing of the manuscript. ÂM and DH wrote the code in R. ÂM developed the interactive web application. All authors contributed to the article and approved the submitted version.

## Conflict of Interest

The authors declare that the research was conducted in the absence of any commercial or financial relationships that could be construed as a potential conflict of interest.

## Footnotes

^1^The discount rate accounts for the decreasing value of benefits and costs over time in “real terms,” as viewed from the present.

^2^More details available here: http://boydorr.gla.ac.uk/eemodel/epiecon.
